# Identification and Expression Analyses of *IL-17/IL-17R* Gene Family in Snakehead (*Channa argus*) Following *Nocardia seriolae* Infection

**DOI:** 10.3390/genes16030253

**Published:** 2025-02-22

**Authors:** Xiufeng Han, Xue Su, Mingyue Che, Lanhao Liu, Pin Nie, Su Wang

**Affiliations:** 1 School of Marine Science and Engineering, Qingdao Agricultural University, 700 Greatwall Road, Qingdao 266109, China; hanxiufeng0401@163.com (X.H.); suxue1423@163.com (X.S.); cmy15942684862@163.com (M.C.); liulanhao@qau.edu.cn (L.L.); 2 Laboratory for Marine Biology and Biotechnology, Qingdao Marine Science and Technology Center, Qingdao 266237, China

**Keywords:** IL-17, IL-17R, snakehead, *Nocardia seriolae*

## Abstract

Background/Objectives: The interleukin 17 (IL-17) family, known for its proinflammatory properties, is important in immune responses against bacterial and fungal infections. To exert its immune function, the IL-17 family typically binds to IL-17 receptor (IL-17R) to facilitate signal transduction. Methods: This study identified, cloned and analyzed seven IL-17 and nine IL-17R family members in snakeheads. Results: A duplication event occurred in snakehead *IL-17s* and *IL-17Rs*, but bioinformatics analyses indicated that these genes were conserved in both protein domains and evolutionary processes. Tissue distribution analysis revealed that *IL-17s*/*IL-17Rs* were widely distributed in the detected tissues, with relatively high expression levels in immune tissues. Upon *Nocardia seriolae* stimulation, most members were expressed, particularly *IL-17C2*, *IL-17D*, *IL-17N*, *IL-17RA1*, *IL-17RA2*, *IL-17RC1*, and *IL-17RE1*, which were significantly upregulated in gill and intestine. Conclusions: These results suggested that *IL-17s* and *IL-17Rs* played a crucial role in mucosal immunity against bacterial infection, providing insights into immunoprophylactic strategies for bacterial diseases in aquaculture.

## 1. Introduction

The interleukin (IL)-17 family exerts a pivotal role in a myriad of biological functions, such as instigating inflammatory cascade during infection and autoimmune disease, as well as bolstering protective immunity against various pathogens [1]. In vertebrates, IL-17s are predominantly secreted by Th17 cells, NK cells, γδ T cells, mast cells, and epithelial cells [2]. IL-17 family customarily binds to IL-17 receptor (IL-17R) to facilitate signal transduction and execute immune functions [3].

In mammals, IL-17 family is composed of six distinct members, namely IL-17A, IL-17B, IL-17C, IL-17D, IL-17E (also designated IL-25) and IL-17F. Their corresponding receptors encompass five members: IL-17RA, IL-17RB, IL-17RC, IL-17RD and IL-17RE [4,5]. IL-17A and IL-17F are both mainly produced by Th17 cells, NK cells, γδ T cells, and share 50% sequence identity [4]. However, IL-17A plays an important role in host defense against bacterial and fungal infections, and IL-17F is mainly involved in host mucosal defense [6]. In a survey of cytokine induction, it was found that IL-17B and IL-17C can stimulate the release of TNFα and IL-1β from monocytic cell line THP-1 [7]. Contemporary research has elucidated that IL-17D, via its receptor CD93, influences the function of group 3 innate lymphoid cells (ILC3s) through regulating intestinal homeostasis [8]. IL-17E primarily induces the secretion of Th2 cytokines and playing a central role in safeguarding against helminth infections [9,10].

In members of IL-17 family, a distinct IL-17 domain is typically positioned at C-terminus. IL-17A and IL-17F, the two prominent members of this family, exhibit comparable protein configurations characterized with four conserved cysteine residues. These residues are essential in disulfide bond formation, which are important for protein three-dimensional architecture and function [6]. All members of IL-17R family possess two extracellular fibronectin II–like (FNIII) domains and one intracellular SEFIR domain, the latter of which resembles TIR domain in terms of length and secondary structure. Proteins containing SEFIR domain likely function as signaling components in pathways similar to Toll/IL-17R [11]. Functional receptors for IL-17 family cytokines consist of homodimers or heterodimers [6]. IL-17A homodimers, IL-17F homodimers, or IL-17A/F heterodimers send signals through IL-17RA/IL-17RC [12,13]. IL-17C signals via IL-17RA/IL-17RE [14], while IL-17E signals through IL-17RA and IL-17RB [15].

Recently, members of *IL-17* and *IL-17R* families have been identified in a few species of teleost, such as black rockfish (*Sebastes schlegelii*), turbot (*Scophthalmus maximus*), spotted sea bass (*Lateolabrax maculatus*), and channel catfish (*Ictalurus punctatus*) [16,17,18,19], However, unlike mammals, teleost lack *IL-17E*, but possess *IL-17N* [16,20,21,22]. Studies have shown that most *IL-17* and *IL-17R* genes are highly expressed in immune-related tissues in teleost [17]. Furthermore, *IL-17* and *IL-17R* genes are upregulated upon stimulation with bacteria or bacterial analogs such as LPS and PGN [18,22].

All recombinant proteins of teleost IL-17s, including IL-17A/F, IL-17B, IL-17C, IL-17D and IL-17N, induce mRNA expression of inflammatory cytokine *TNF-α*, as well as chemokine *CXCL8* [22,23,24,25]. Among these IL-17s, only IL-17A/F and IL-17N can stimulate the expression of the antimicrobial peptide S100A [22,25]. IL-17B, C and D can enhance phagocytosis and chemotaxis of leukocytes [23]. Interestingly, *IL-17s* expressions are downregulated following viral stimulation [20]. Similarly, expressions of *IL-17* receptors are suppressed after parasite infection [26].

Snakehead (*Channa argus*) is a valuable economic freshwater fish species in China, with annual production exceeding 605,438 tons in 2023 [27]. However, cultured snakeheads are severely affected by Gram-positive bacterial pathogen *N*. *seriolae*. This pathogen causes white nodules in internal organs and skin ulceration, and leads to mortality that can reach up to 100% post-onset, resulting in substantial economic losses to aquaculture industry [28,29]. Compared to other aquaculture fish like turbot (*S*. *maximus*) and spotted sea bass (*L*. *maculatus*) [17,18,30,31], less research is reported on immune-related genes and immune responses to pathogens in snakeheads. Therefore, investigating immune-related genes, including *IL-17s/IL-17Rs*, and their functions in the immune response of snakeheads during pathogen infection is urgent. This study aims to assess the expression patterns of snakehead *IL-17s/IL-17Rs* genes upon *N*. *seriolae* infection. To accomplish this objective, a genome-wide identification of *IL-17s/IL-17Rs* in snakeheads were conducted. Further analyses were employed on their molecular characteristics and phylogenetic relationships. Subsequently, tissue expression patterns of *IL-17/IL-17R* mRNAs in healthy snakeheads, as well as in those infected with *N. seriolae*, were examined and analyzed. This systematic investigation of *IL-17/IL-17R* gene family in snakeheads may contribute to understanding the immune response against *N*. *seriolae*, thereby offering insights into innate immune mechanisms of host defense against bacteria.

## 2. Materials and Methods

### 2.1. Identification of Snakehead IL-17/IL-17R Genes

To acquire all possible *IL-17/IL-17R* genes in snakeheads, two methods were employed. Sequences of snakehead *IL-17/IL-17R* family genes were identified by searching the whole genome using TBLASTN (1 × 10^−5^) (https://www.ncbi.nlm.nih.gov/ (accessed on 20 December 2022)). The available IL-17/IL-17R sequences from other species were listed in Table S1. Additionally, HMMER search tool and Pfam database (http://pfam.xfam.org/family/ (accessed on 20 December 2022)) were utilized to search for the highly conserved C-terminus domains of IL-17 and IL-17R, such as IL-17 domain and SEFIR domain (Pfam-B_9152 and Pfam-B_33671) [32]. Subsequently, the coding sequence (CDSs) of all snakehead *IL-17/IL-17R* genes were cloned. Primers used for gene cloning were shown in Table S2. Afterwards, BLASTN and BLASTP were used to further confirm the sequence authenticity. Theoretical isoelectric point (pI) and molecular weight (MW) of snakehead IL-17/IL-17R proteins were computed by ExPASy server (https://web.expasy.org/compute_pi/ (accessed on 20 December 2022)).

### 2.2. Bioinformatics Analysis of Snakehead IL-17s/IL-17Rs

Phylogenetic analyses were conducted to confirm the annotation of these identified snakehead IL-17/IL-17R proteins. Amino acid sequences of IL-17/IL-17R from other vertebrates were downloaded from the NCBI database to construct phylogenetic trees. Sequences used here were listed in Table S1. Multiple amino acid sequences were aligned using ClustalW2 program (https://www.ebi.ac.uk/Tools/msa/clustalw2/ (accessed on 20 December 2022)) with default parameters. ML phylogenetic tree was built by MEGA 7 software with 1000 replicate bootstrap test [33].

Snakehead IL-17/IL-17R protein domains were predicted with SMART website (http://smart.embl-heidelberg.de (accessed on 20 December 2022)) and exon-intron structure were generated by Splign (https://www.ncbi.nlm.nih.gov/sutils/splign/splign.cgi (accessed on 20 December 2022)). Amino acid similarity of snakehead IL-17/IL-17R proteins were compared by MegAlign (DNAstar, Madison, WI, USA).

### 2.3. Fish Sampling and Challenge Experiment

The School of Marine Science and Engineering at Qingdao Agricultural University approved all experiments (QAU-SMSE-202211), and we adhered to the general guidelines for the care and use of laboratory animals.

Snakeheads used were purchased from Neijiang County, Sichuang Province, and each fish weighted about 100 g ± 10 g. A total of 18 healthy experimental snakehead fish were raised in a cylindrical culture tank with a diameter of 120 cm and a height of 75 cm (actual water depth: 65 cm) in the laboratory for one month. During the fish-farming process, temperature was maintained at 28 °C and dissolved oxygen level was kept at 5–6 mg/L. Feeding (purchased from Tongwei, China) was terminated one day prior to experiments. Liver, spleen, head kidney, intestine, skin, muscle, gill, and brain were sampled from three healthy fish, and frozen in liquid nitrogen immediately for RNA extraction. During sampling, fish were initially anesthetized with MS-222 (100 mg/L).

*N. seriolae* was isolated from diseased snakeheads before, and was cultured in a brain–heart infusion broth at 28 °C. Snakeheads were infected by *N. seriolae* (1 × 10^7^ CFU/mL) for 2 h in a 30 L (18 L water) aquaria. Subsequently, the treated fish were transferred back to freshwater. Other uninfected, healthy fish served as control. Samples were collected at 0 h (as control), 12 h, 24 h, 48 h, and 72 h post challenges. At each sampling point, samples were collected from three fish. For sampling, fish from infected or control groups were euthanized with MS-222 (100 mg/L). Due to gill, intestine, and skin being important mucosal immune organs in teleost [34], these tissues were collected and frozen immediately in liquid nitrogen to be prepared for RNA extraction.

### 2.4. RNA Extraction and Quantitative Real-Time PCR Analyses (qPCR)

Total RNA was extracted using TRIzol^®^ reagent (Invitrogen, Carlsbad, CA, USA) according to the manufacturer’s protocol. Concentration and quality of these RNA was detected by gel electrophoresis apparatus (Liuyi, Beijing, China) and ultramicrospectrophotometer (DeNovix, Wilmingon, DE, USA). 1 μg RNA of each sample was reversed transcribed into cDNA by HiScript III RT SuperMix for qPCR (+gDNA wiper) (Vazyme, Nanjing, China). Primers of snakehead IL-17/IL-17R genes were designed using Primer5 software (Table S2). The primer specificity amplification was verified by melting curve and agarose gel electrophoresis.

qRT-PCR was performed using Applied Biosystems QuantStudio 5 (Applied Biosystems, Waltham, MA, USA) and 2 × SYBR Green qPCR Mix (With ROX) (Sparkjade, Qingdao, Shandong, China). The total 20 μL experimental volume included 1 μL cDNA, 10 μL SYBR Green qPCR Mix (2×), 0.8 μL primers, 0.4 μL ROX Reference Dye and 7.8 μL RNase-free H_2_O. qRT-PCR analysis was carried out using the following conditions: 94 °C for 3 min, 40 cycles of 94 °C for 20 s and 62 °C for 20 s, followed by 72 °C for 30 s. Relative expression levels of RNA were calculated using the comparative 2^−ΔΔCT^ method, and *β-actin* of snakehead was set as internal reference gene. The experiments were performed triple times for technical and biological repetitions. All of the qPCR experiments were conducted according to the MIQE guidelines [35].

### 2.5. Statistical Analysis

Data were presented as mean ± standard error (SE). The statistical analyses were conducted using SPSS 27.0 software (IBM, Chicago, MA, USA). One-way analysis of variance (ANOVA) was employed to analyze the experimental data. Differences were considered to be significant at *p* < 0.05.

## 3. Results

### 3.1. Identification and Characterization of IL-17/IL-17R Genes in Snakehead

Seven *IL-17* genes, including *IL-17A/F1*, *IL-17A/F2*, *IL-17A/F3*, *IL-17C1*, *IL-17C2*, *IL-17D* and *IL-17N* (accession number were listed in Table 1) were identified in the available database of snakehead genome (ASM478618v1). Length of the CDS of these identified *IL-17* genes ranged from 399 to 645 bp, encoding peptides of 132 to 214 aa, with molecular weighting being 14.69 to 24.04 kDa. There were two exons in *IL-17D* and *IL-17N*, three exons in *IL-17A/F1*, *IL-17A/F2*, *IL-17C1* and *IL-17C2*, four exons in *IL-17A/F3*. Isoelectric point (pI) value of these molecules varied from 6.17 to 10.26 (Table 1).

Nine *IL-17R* genes were also found in the snakeheads, and annotated as *IL-17RA1*, *IL-17RA2*, *IL-17RB*, *IL-17RC1*, *IL-17RC2*, *IL-17RD1*, *IL-17RD2*, *IL-17RE1*, and *IL-17RE2* (accession number were listed in Table 1). Their CDS spanned from 1314 to 2442 bp in length, encoding proteins of 437 to 813 aa, and had molecular weights ranging 49.27 to 91.74 kDa. Snakehead *IL-17Rs* consisted of 10 to 18 exons and their pI values varied from 5.18 to 9.05 (Table 1).

**Figure 1:**
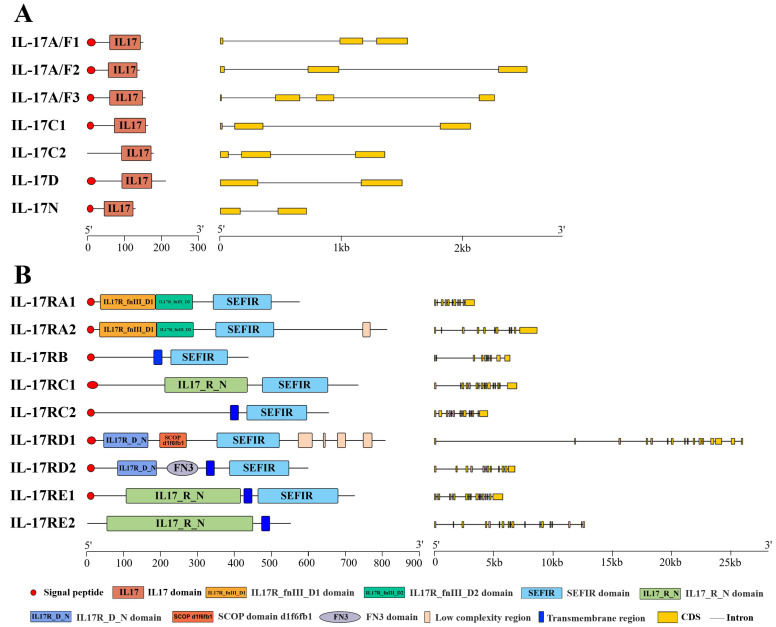
Conserved domains and exon/intron structures of IL-17s (**A**) and IL-17Rs (**B**) in snakeheads. The domains were represented by different colors and shapes. Yellow boxes represented exons. The length of protein and gene sequence could be inferred by the scale.

These identified sequences of snakehead IL-17s and IL-17Rs were then analyzed in both sequence identity and protein domains. Sequence alignment showed that IL-17s shared a rather low identity (ranging from 19.5% to 40.8%), so did IL-17Rs (ranging from 6.8% to 45.2%) (Figure S1). However, their protein domains were conserved. All IL-17s possessed a signal peptide (except IL-17C2) and a typical IL-17 domain (Figure 1A). Excluding IL-17RE2, IL-17Rs existed a signal peptide and a common SEFIR domain. With the exception of *IL-17RB*, a gene duplication event occurred in all snakehead *IL-17Rs*. Duplicated IL-17RA (IL-17RA1 and IL-17RA2) possessed an IL17R_fnIII_D1 and IL17R_fnIII_D2 domain. An IL17_R_N domain was present only in IL-17RC1, IL-17RE1 and IL-17RE2. IL-17RD1 and IL-17RD2 owned an IL17R_D_N domain, while the latter one had an extra fibronectin-3 (FN3)-like domain (Figure 1B).

### 3.2. Phylogenetic Analyses of IL-17s/IL-17Rs

**Figure 2:**
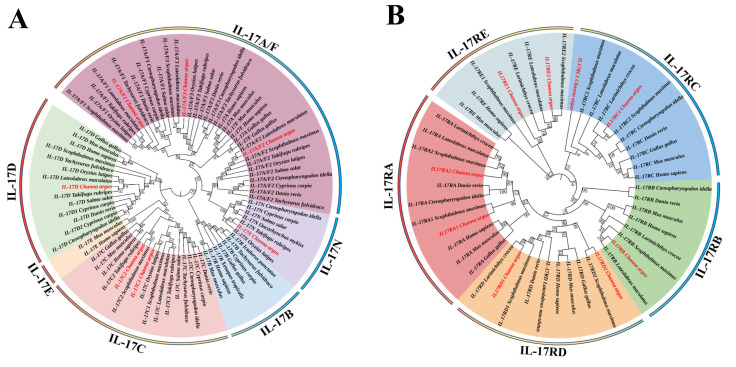
Phylogenetic relationships of IL-17s (**A**) and IL-17Rs (**B**) from the snakehead and other selected species. Phylogenetic trees were constructed using the neighbor-joining (NJ) method with 1000 bootstrap replications using MEGA 7. Subfamily genes were marked with different colors. IL-17/IL-17R family member of snakehead was marked in red font. GenBank accession numbers of IL-17 and IL-17R amino acid sequences used here were listed in Table S1.

To confirm our annotations and the evolutionary relationship of IL-17s/IL-17Rs in snakehead, two phylogenetic trees were constructed. Overall, all snakehead IL-17s were clustered with their counterparts in other vertebrates, respectively. In teleost, there was a lineage-specific gene IL-17N, duplicated IL-17Cs, and three IL-17A/Fs. Teleost IL-17A/F2 were clustered with IL-17N into one branch, while teleost IL-17A/F1 and IL-17A/F3 showed a closer relationship with IL-17A or IL-17F in other vertebrates. Moreover, IL-17B was absent in both snakehead and spotted sea bass (*L. maculatus*) and turbot (*S. maximus*), contrasting with its presence in channel catfish (*I. punctatus*), common carp (*Cyprinus carpio*) and fugu rubripes (*Tachysurus fulvidraco*). To date, IL-17E is reported only in mammals (Figure 2A). All IL-17Rs in snakehead were gathered with their vertebrate homologues into five separate clades (IL-17RA, IL-17RB, IL-17RC, IL-17RD, and IL-17RE) (Figure 2B).

### 3.3. Tissues Distribution of Snakehead IL-17/IL-17R Genes

**Figure 3:**
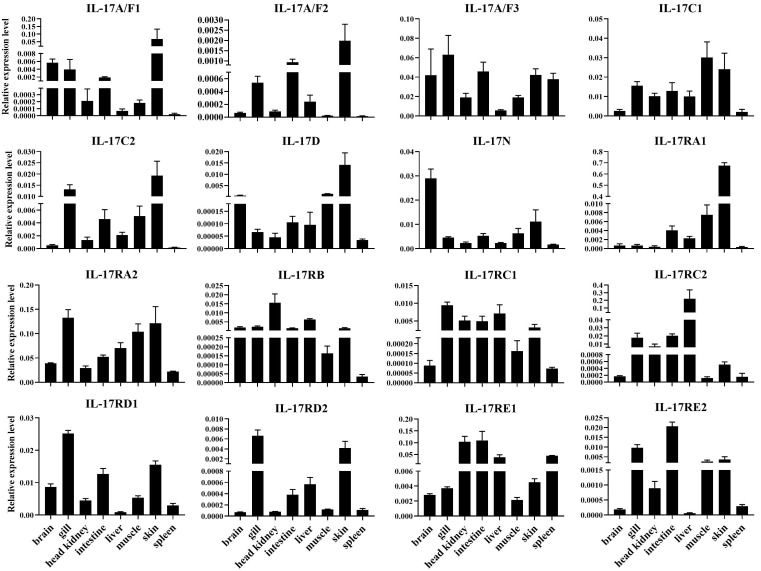
Expression pattern of *IL-17s/IL-17Rs* in various tissues of healthy snakeheads. The relative expressions of *IL-17/IL-17R* mRNAs were normalized to *β-actin*. The data were shown as mean ± SEM (n = 3).

Snakehead *IL-17/IL-17R* gene expression levels were measured in eight tissues from healthy fish, such as liver, spleen, head kidney, intestine, skin, muscle, gill, and brain. As shown in Figure 3, *IL-17s* and *IL-17Rs* had low expression levels in all examined tissues. Interestingly, these genes showed tissue-specific distribution, especially in immune related tissues. *IL-17A/F2*, *IL-17A/F3*, *IL-17C2*, *IL-17RA1*, *IL-17RA2*, *IL-17RB*, *IL-17RC1*, *IL-17RC2*, *IL-17RD1*, *IL-17RD2*, *IL-17RE1*, and *IL-17RE2* were expressed in relatively high levels in intestine, skin, gill, head kidney, liver, or spleen. However, other *IL-17s* such as *IL-17A/F1*, *IL-17A/F3*, and *IL-17D* exhibited high expression levels in brain, and the teleost-specific gene *IL-17N* also had a high expression in the brain.

### 3.4. Expression Patterns of Snakehead IL-17s/IL-17Rs After N. seriolae Infection

**Figure 4:**
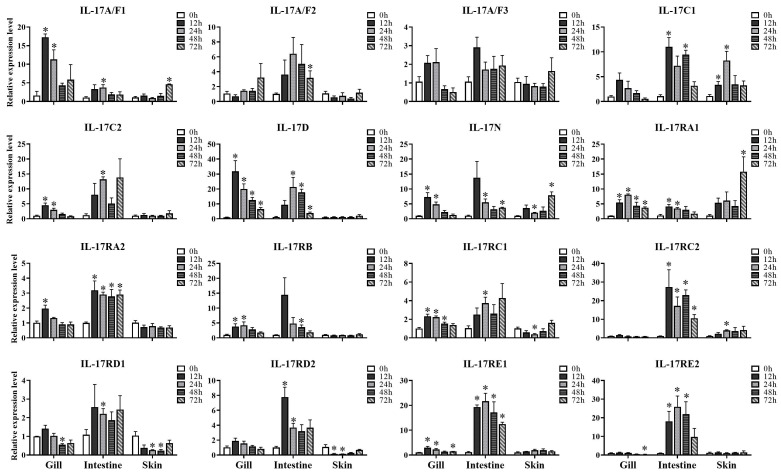
Expression levels of *IL-17/IL-17R* genes in gills, intestines, and skin were measured at different time points (12 h, 24 h, 48 h and 72 h) after *N. seriolae* infection, with 0 h as control. The results were presented as the mean ± SE of fold changes. One-way ANOVA was conducted to assess the variability between the experimental and control groups (* *p* < 0.05).

*N. seriolae* was used to examine the response of *IL-17* and *IL-17R* genes to bacterial infection. The expression patterns of *IL-17s* and *IL-17Rs* were subsequently examined in skin, gills, and intestines at 0, 12, 24, 48, and 72 h post infection (hpi) (Figure 4).

In the gills, *IL-17A/F1*, *IL-17C2*, *IL-17D*, *IL-17N*, *IL-17RA2*, *IL-17RC1*, and *IL-17RE1* exhibited similar expression patterns, achieving their highest levels at 12 hpi, after which a decrease was observed. But there was a disparity in the expression patterns of *IL-17RA1* and *IL-17RB*, with both being upregulated at 12 hpi, peaking at 24 hpi, and then decreasing. Unlike the others, *IL-17RD1* and *IL-17RE2* showed no significant difference, except for a marked downregulation at 48 hpi for *IL-17RD1* and 72 hpi for *IL-17RE2*.

In the intestines, all *IL-17s* and *IL-17Rs*, except *IL-17A/F3*, had obvious upregulation following *N. seriolae* infection. But this increase happened at various time points like 12 hpi (*IL-17C1*, *IL-17RA1*, *IL-17RA2*, *IL-17RC2*, *IL-17RD2*, *IL-17RE1*, *IL-17RE2*), 24 hpi (*IL-17A/F1*, *IL-17C2*, *IL-17D*, *IL-17N*, *IL-17RC1*, *IL-17RD1*), 48 hpi (*IL-17RB*), and 72 hpi (*IL-17A/F2*). And the duration of this upregulation state varied from 12 to 72 h.

In the skin, significant elevation was observed at 72 hpi for *IL-17A/F1*, between 24 and 48 hpi for *IL-17C1*, at both 24 and 72 hpi for *IL-17N*, at 72 hpi for *IL-17RA1* and at 24 hpi for *IL-17RC2*. Notably, downregulation was the predominant trend detected at 24 hpi for *IL-17RC1*, between 24 and 48 hpi for *IL-17RD1* and between 12 and 24 hpi for *IL-17RD2*. Other *IL-17s* and *IL-17Rs* did not show no changes before or after infection.

## 4. Discussion

In this study, we identified and cloned seven IL-17 genes (IL-17A/F1, IL-17A/F2, IL-17A/F3, IL-17C1, IL-17C2, IL-17D, and IL-17N) and nine IL-17R genes (IL-17RA1, IL-17RA2, IL-17RB, IL-17RC1, IL-17RC2, IL-17RD1, IL-17RD2, IL-17RE1, and IL-17RE2) in snakeheads.

The present study has identified structural domains is conserved in both IL-17 and IL-17R proteins, like IL-17 domain and SEFIR domain [36,37]. A conserved IL-17 domain and a signal peptide were found in all snakehead IL-17s, while the latter one was lacked in IL-17C2. And IL-17Rs in snakeheads, except for IL-17RE2, had a typical SEFIR domain. This agreed with the findings in black rockfish (*S. schlegelii*) and turbot (*S. maximus*) [16,17]. SEFIR domain was believed to be necessary for downstream signaling pathways [5], so it was supposed snakehead IL-17RE2 and other IL-17Rs might differ in signal transduction. Both IL-17RA1 and IL-17RA2 comprised two fibronectin-3-like (FN3) domains, namely IL-17R_fnIII_D1 and IL-17R_fnIII_D2, which was consistent with mammalian research [1]. Furthermore, IL-17RC1, IL-17RE1 and IL-17RE2 were identified to possess an IL-17_R_N domain at N-terminus, while IL-17RD1 and IL-17RD2 owned an IL-17R_D_N domain. These existed conserved motifs meant that they might exert similar functions.

Similarly to the previous reports on teleosts [16,17,18,19,20,25,38,39,40], the snakehead has three repeats of *IL-17A/F*, and two repeats of *IL-17C*, *IL-17RA*, *IL-17RC*, *IL-17RD,* and *IL-17RE*. Among them, *IL-17N* is a teleost-specific gene that was first identified in Japanese pufferfish (*Takifugu rubripes*) and has since been found only in other teleosts [41,42]. It was clustered with IL-17A/F closely, suggesting they might share an ancestral gene.

Gene duplication is considered as a significant event for gene family expansion, and teleosts undergo third genome duplication [43,44]. The results of gene syntenic analyses of *IL-17/IL-17R* showed that in human (*Homo sapiens*), chicken (*Gallus gallus*) and African clawed frogs (*Xenopus laevis*), the *IL-17A* and *IL-17F* genes were found to be tandemly arranged. Similarly, in zebrafish (*Danio rerio*) and tilapia (*Oreochromis niloticus*), the *IL-17A/F1* and *IL-17A/F2* genes were also tandemly organized [22]. Furthermore, in zebrafish (*D. rerio*) and stickleback (*Gasterosteidae aculeatus*), two *IL-17RA* genes were found to be tandemly arranged [45]. Gene duplication event could explain why teleosts possessed multiple copies of *IL-17* and *IL-17R* genes. Despite gene duplication events in snakehead genome that may generate multiple variants of *IL-17s* and *IL-17Rs*, their expression patterns varied. As depicted in this study, the duplicated *IL-17A/F*, *IL-17C*, *IL-17RA*, *IL-17RC*, *IL-17RD* and *IL-17RE* demonstrated both similar but distinct expression profiles. These findings coincided with the theory that duplicated genes may perform similar, complementary or divergent functions, which should be the subject of future studies [46,47].

Results in our study revealed that snakehead *IL-17s/IL-17Rs* were widely expressed in all tissues. Typically, high expression levels of these *IL-17s/IL-17Rs* were found in immunological relevant tissues such as skin, gill, intestines, head, kidneys and liver. It was consistent with observations in other teleost [16,17,23]. The distribution of *IL-17/IL-17Rs* in immune organs may imply their potential role in innate immune responses of teleost. Notably, studies have shown that *IL-17N*, a cytokine unique to teleost, predominantly exhibited a strong expression in brain. This phenomenon had been documented across different teleosts, including black rockfish (*S. schlegelii*) [16], common carp (*C. carpio*) [21], yellow catfish (*Pelteobagrus fulvidraco*) [22] and European sea bass (*Dicentrarchus labrax*) [20], highlighting its possible role in the central nervous system’s immune function in these aquatic vertebrates.

Teleost mucosal epithelial barriers, such as gill, intestine and skin play immune roles in defending against pathogens [48,49,50,51]. In this study, the general upregulation of snakehead *IL-17s* and *IL-17Rs* were detected in gills, skin, and intestines after *N. seriolae* infection. These patterns were consistent with the findings in common carp (*C*. *carpio*), Asian swamp eel (*Monopterus albus*), channel catfish (*I*. *punctatus*) and yellow catfish (*P*. *fulvidraco*) [19,22,23,24,52]. Previous studies have shown *IL-17A/F*, *IL-17B*, *IL-17C*, *IL-17D* and *IL-17N* could stimulate the expression of proinflammatory cytokines, chemokines and antimicrobial peptides in response to bacterial infection [22,23,41]. This mechanism may be attributed to IL-17 regulating signaling pathways such as NF-κB and MAPK [22,39]. These results may indicate *IL-17s* and *IL-17Rs* were activated in mucosal immune responses and imply the existence of complex immune regulatory mechanisms against bacterial invasion.

In snakehead, nearly all *IL-17s* and *IL-17Rs* were significantly elevated in intestine, albeit at various time points, suggesting distinct regulatory mechanisms in intestinal immunity. As previously reported, Japanese medaka (*Oryzias latipes*) with IL-17RA1 deficiency exhibited dysregulated intestinal microbiota, with an increase in conditional pathogenic bacteria observed [53]. *IL-17A/F1* KO in *O. latipes* led to dysregulated intestinal microbiota and impaired immune gene expression [54]. Therefore, we propose that *IL-17s* and *IL-17Rs* of snakeheads play key roles in intestinal immune response against bacterial infection.

## 5. Conclusions

In this study, seven *IL-17* family members and nine *IL-17* receptor family members were identified and cloned in snakehead. A duplication event occurred in snakehead *IL-17s* and *IL-17Rs*, but bioinformatics analyses indicated that these genes were conserved in both protein domains and evolutionary processes. Tissue distribution analysis revealed that *IL-17s/IL-17Rs* were widely distributed in the detected tissues, with relatively high expression levels in immune tissues. After stimulation by *N. seriolae*, most of *IL-17s* and *IL-17Rs* genes exhibited a significant response to the infection but in various expression patterns, indicating regulatory differences in their function against bacterial infection. In addition, these obvious upregulation of snakehead *IL-17s* and *IL-17Rs* in gill and intestine upon infection, suggesting their crucial role in mucosal immunity. However, the presence of multiple IL-17s and IL-17Rs in snakehead meant the binding patterns between ligands and receptors could thus generate more possibilities [41]. Therefore, the binding combination and regulatory mechanisms of IL-17 ligand and receptor in teleosts are still in need of further investigation.

## Figures and Tables

**Table 1:** Identification of *IL-17/IL-17R* genes identified from snakehead.

Gene Name	CDS Length (bp)	Number of AMINO Acids	Exon Number	Molecular Weight (kDa)	Isoelectric Point	Accession Number
*IL-17A/F1*	462	153	3	16.78	8.69	PQ625371
*IL-17A/F2*	432	143	3	15.67	8.14	PQ625372
*IL-17A/F3*	480	159	4	17.36	10.26	PQ625373
*IL-17C1*	498	165	3	18.61	8.31	PQ625374
*IL-17C2*	546	181	3	21.06	9.57	PQ625375
*IL-17D*	645	214	2	24.04	9.80	PQ625376
*IL-17N*	399	132	2	14.69	6.17	PQ625377
*IL-17RA1*	1731	576	11	66.11	5.97	PQ625378
*IL-17RA2*	2442	813	11	91.74	5.18	PQ625379
*IL-17RB*	1314	437	10	49.27	8.78	PQ625380
*IL-17RC1*	2208	735	16	81.21	7.97	PQ625381
*IL-17RC2*	1968	655	14	74.96	6.61	PQ625382
*IL-17RD1*	2427	808	16	89.77	6.80	PQ625383
*IL-17RD2*	1800	599	12	67.10	6.33	PQ625384
*IL-17RE1*	2178	725	17	81.37	9.05	PQ625385
*IL-17RE2*	1659	552	18	61.48	6.57	PQ625386

## Data Availability

The original contributions presented in the study are included in the article, further inquiries can be directed to the corresponding author.

## Supplementary Materials

The following supporting information can be downloaded at: https://www.mdpi.com/article/10.3390/genes16030253/s1, Table S1: The amino acid sequence Accession number used to TBLSTN and construct the phylogenetic tree; Table S2: Primers used in this study; Figure S1: Amino acid similarity of snakehead IL-17 (A) and IL-17R (B) proteins.
