# Supplementary material for: Identification and Expression Analyses of IL-17/IL-17R Gene Family in Snakehead (Channa argus) Following Nocardia seriolae Infection
Source: Genes (Basel). 2025 Feb 22;16(3):253. doi: 10.3390/genes16030253 (PMC11942210; doi:10.3390/genes16030253)
Supplement: Supplementary file 1 [file genes-16-00253-s001.zip › genes-3373401-supplementary.pdf]

Table S1. The amino acid sequence Accession number used to TBLSTN and construct the phylogenetic tree.

Species name	Protein name	Accession no.
Danio rerio	IL-17A/F1	NP_001018623.1
	IL-17A/F2	NP_001018634.1
	IL-17A/F3	NP_001018626.1
	IL-17C	NP_001018624.1
	IL-17D	NP_001018625.1
	IL-17RA	NP_001093473.1
	IL-17RB	XP_056320539.1
	IL-17RC	XP_009304517.1
	IL-17RD	NP_705946.3
Ictalurus punctatus	IL-17B	XP_017341329.1
Ctenopharyngodon idella	IL-17A/F1	XP_051724618.1
	IL-17A/F2	XP_051724613.1
	IL-17A/F3	XP_051732252.1
	IL-17C	XP_051726051.1
	IL-17D	XP_051761592.1
	IL-17N	XP_051751258.1
	IL-17RA	AXF94778.1
	IL-17RB	AXF94779.1
	IL-17RC	AXF94780.1
Scophthalmus maximus	IL-17A/F1	XP_035483674.2
	IL-17A/F2	XP_035483680.1
	IL-17A/F3	XP_035474391.1
	IL-17C1	XP_035498339.2
	IL-17C2	XP_035484926.1
	IL-17D	XP_035507122.1
Salmo salar	IL-17A/F1	XP_014048030.2
	IL-17A/F2	XP_014049021.1
	IL-17A/F3	AJF46588.1
	IL-17RA	AXF94778.1
	IL-17RB	AXF94779.1

	IL-17RC	AXF94780.1
	IL-17C	XP_013980235.1
	IL-17D	NP_001134365.1
	IL-17N	AJF46589.1
Takifugu rubripes	IL-17A/F1	BAI82578.1
	IL-17A/F2	XP_011608062.2
	IL-17A/F3	XP_011610026.1
	IL-17C1	BAI82581.2
	IL-17C2	BAI82582.2
	IL-17D	XP_003962280.2
	IL-17N	BAI82584.1
	IL-17N	AJF46594.1
Lateolabrax maculatus	IL-17A/F1	QRW33321.1
	IL-17A/F2	QRW33322.1
	IL-17A/F3	QRW33323.1
	IL-17C	QRW33324.1
	IL-17D	QRW33325.1
	IL-17RA	QRW33326.1
	IL-17RB	QRW33327.1
	IL-17RC	QRW33328.1
	IL-17RD	QRW33329.1
	IL-17RE	QRW33330.1
Tachysurus fulvidraco	IL-17A/F1	XP_047677716.1
	IL-17A/F2	XP_026990552.2
	IL-17A/F3	XP_027019136.2
	IL-17B	XP_027028871.1
	IL-17C	XP_027017664.1
	IL-17D	XP_027020144.2
Gallus gallus	IL-17A	NP_989791.2
	IL-17B	XP_040503070.1
	IL-17C	XP_003641993.2
	IL-17D	ENSGALT00010013234.1
	IL-17F	XP_426223.4

	IL-17RA	NP_001305917.2
	IL-17RC	XP_015148828.2
	IL-17RD	XP_040502003.1
Cyprinus carpio	IL-17A/F1	XP_042629596.1
	IL-17A/F2	BAV01250.1
	IL-17B	XP_018954810.1
	IL-17C	WDK97535.1
	IL-17D1	BAV01254.1
	IL-17D2	BAV01255.1
	IL-17N	BAV01251.1
	IL-17RA1	BAV01257.1
	IL-17RA2	BAV01258.1
	IL-17RB	BAV01259.1
	IL-17RC1	BAV01260.1
	IL-17RC2	BAV01261.1
	IL-17RD1	BAV01262.1
	IL-17RD2	BAV01263.1
	IL-17RE1	BAV01264.1
	IL-17RE2	BAV01265.1
Oryzias latipes	IL-17A/F1	NP_001191714.1
	IL-17A/F2	XP_020557054.1
	IL-17A/F3	NP_001191715.1
	IL-17C	NP_001191723.1
	IL-17D	XP_011487735.1
	IL-17N	NP_001191717.1
	IL-17RA	NP_055154.3
	IL-17RB	NP_061195.2
	IL-17RC	NP_703191.2
	IL-17RD	NP_060033.3
	IL-17RE	NP_705616.2
Homo sapiens	IL-17A	NP_002181.1
	IL-17B	CAG33473.1
	IL-17C	NP_037410.1

	IL-17D	AAQ89471.1
	IL-17E	NP_073626.1
	IL-17F	NP_443104.1
	IL-17RA	NP_055154.3
	IL-17RB	NP_061195.2
	IL-17RC	NP_703191.2
	IL-17RD	NP_060033.3
	IL-17RE	NP_705616.2
Mus musculus	IL-17A	NP_034682.1
	IL-17B	NP_062381.1
	IL-17C	NP_665833.4
	IL-17D	NP_665836.2
	IL-17E	NP_542767.1
	IL-17F	NP_665855.2
	IL-17RA	NP_032385.1
	IL-17RB	NP_062529.2
	IL-17RC	NP_598920.3
	IL-17RD	NP_602319.1
	IL-17RE	NP_665825.2
Scophthalmus maximus	IL-17RA1	XP_035497818.2
	IL-17RA2	XP_035480972.2
	IL-17RB	XP_035499898.1
	IL-17RC1	XP_035500782.2
	IL-17RC2	XP_035487428.2
	IL-17RD1	XP_035486025.2
	IL-17RD2	XP_035501440.1
	IL-17RE1	XP_035500785.1
	IL-17RE2	XP_035469993.1
Larimichthys crocea	IL-17RA	APM87153.1
	IL-17RB	APM87154.1
	IL-17RC	APM87155.1
	IL-17RD	APM87156.1
	IL-17RE	APM87157.1

Table S2. Primers used in this study.

Primer name	Sequence(5'-3')	Application
IL-17A/F1 ORF F	ATGTTTTCATCCAAAATGATGGTTG	**Gene clone**
IL-17A/F1 ORF R	TTATTTTTGGTGTAGGATGTTGGGT	
IL-17A/F2 ORF F	ATGAAGCTGAGAATTGACGTCTTCG	
IL-17A/F2 ORF R	TCAGTTTTGGCTGATTTTGGCCC	
IL-17A/F3 ORF F	ATGCTGCTGGTACTGAGAGCTCTGT	
IL-17A/F3 ORF R	TTACCGCTGTGATATGACACTGGG	
IL-17C1 ORF F	ATGGACATGAAGCAGATTCTCATAT	
IL-17C1 ORF R	TTACGAGGAGGTCTGGACTCTG	
IL-17C2 ORF F	ATGGAGCGGTCGGTCACAGTCA	
IL-17C2 ORF R	TTATTTCTTAGCATACTTGGGCACAACG	
IL-17D ORF F	ATGCCGCGTCGGATCCG	
IL-17D ORF R	TCACAGCCCTACTTTCTTCACTTTCTTG	
IL-17N ORF F	ATGATGCGAGTCTTTCTGCTGTG	
IL-17N ORF R	TCATGTTGAGCTGTGCCTGGAAA	
IL-17RA1 ORF F	ATGTGCCGTGTTGCTTTTTTCT	
IL-17RA1 ORF R	TTACTCCTGGATAGATGGATAG	
IL-17RA2 ORF F	ATGAGCTTTTTCCGAGCCG	
IL-17RA2 ORF R	TTAACTCTCTTCAGGGCCTTTATTG	
IL-17RB ORF F	ATGATGTGGGGAGTCACGTTGATG	
IL-17RB ORF R	TCAAATAGAGTTGGCCACACATTGTACT	
IL-17RC1 ORF F	ATGGCTCCAACGCGTCTAC	
IL-17RC1 ORF R	TTAGACCCATTTCATTTCATTGACTTT	
IL-17RC2 ORF F	ATGTTTCCGGGATGGTCAT	
IL-17RC2 ORF R	TCACGGTTGCTGTAAGAACTTT	
IL-17RD1 ORF F	ATGGCGGACTCTCCAAGTTTCTTC	
IL-17RD1 ORF R	CTACAACGATGCTACAGGAGTGAGT	
IL-17RD2 ORF F	ATGTGGAGGGCAGCACTTGTTTTC	
IL-17RD2 ORF R	TTAACCCTCCACAGAGCGAAGCAT	
IL-17RE1 ORF F	ATGCGCACCGCGCTGG	
IL-17RE1 ORF R	TCAGCCCTTGCCTTTTATTTGAGG	
IL-17RE2 ORF F	ATGATTCTCTGGGTGGCCTTACTGA	
IL-17RE2 ORF R	TCAGTCTGATATTAAATTGGCCTTCTCTG	
IL-17A/F1 qPCR F	TCTCTGTTCCCTCCAGTACTGTC	**qRT-PCR**
IL-17A/F1 qPCR R	GAGACTCAAGCCTGTAGTGGTAA	
IL-17A/F2 qPCR F	GAGACCGACACAACCTGAACTC	
IL-17A/F2 qPCR R	GCCACAGACTTGTATGATGCAAT	
IL-17A/F3 qPCR F	TGTGTGCCGTCTCGGTTGC	
IL-17A/F3 qPCR R	CCTGGTAGTGGATGGGTTT	
IL-17C1 qPCR F	CTACAACTCGGTCCCCGTAATGC	
IL-17C1 qPCR R	GTACAGCCCACAGCTACTTCCACA	
IL-17C2 qPCR F	ACCTCAATGACAGCAGGTACCCTC	
IL-17C2 qPCR R	TGACCACCAACTGAACAAACACAA	

IL-17D qPCR F	TCTACGCTCCCTCTGTCAT
IL-17D qPCR R	GTCGCCTCGCTCTTTCTCC
IL-17N qPCR F	GCGAGTCTTTCTGCTGTGTC
IL-17N qPCR R	ACGTAGCTCCATGTTGCGAT
IL-17RA1 qPCR F	TATCATCAGTGCCTCTCA
IL-17RA1 qPCR R	CTCCTCAACATCCAACTC
IL-17RA2 qPCR F	TCCGAGCCGTTGTGCTGTTGCT
IL-17RA2 qPCR R	CTGTCTCATCCTGCCTGGTGTC
IL-17RB qPCR F	GCCATTGGAACTGAGAGCCT
IL-17RB qPCR R	TCCCCGGTCTGAAGAACAGA
IL-17RC1 qPCR F	GTGTTAGTCCTCACCCAGGC
IL-17RC1 qPCR R	TCTGCGAGAATGCAGCTCAA
IL-17RC2 qPCR F	TCAGAGTTAATTTGTCGGTTCGG
IL-17RC2 qPCR R	GTTTTGTTCAAGGCAGTGTGGTT
IL-17RD1 qPCR F	TCTTCACCTCTCTCTCCGGG
IL-17RD1 qPCR R	CTGAAGGTGACGGCCAATCT
IL-17RD2 qPCR F	AATGTATCAGACAGGGAGGACC
IL-17RD2 qPCR R	TTTCCAAGCAACTCAAAACACG
IL-17RE1 qPCR F	GGAGCAGCAGGTTGGCTTTAT
IL-17RE1 qPCR R	AGTCCTGGTTTGTCTTTGTGAGC
IL-17RE2 qPCR F	ATTTGTTCTCCCCCCCAT
IL-17RE2 qPCR R	GCACTTCTGTTTTCCCTCAC
β-actin qPCR F	GAGTGTGCGCATCTACGAG<br>CACTGTGCCCATCTACGAG
β-actin qPCR R	GCATCTCCTGCTCGAAGTC<br>CCATCTCCTGCTCGAAGTC

A

Percent Identity

Divergence	1	2	3	4	5	6	7		
1		26.4	40.8	23.7	25.9	28.7	21.7	1	IL-17 A/F1
2	100.0		29.6	29.8	21.8	32.3	28.8	2	IL-17 A/F2
3	100.0	100.0		27.4	25.2	31.6	25.0	3	IL-17 A/F3
4	100.0	100.0	100.0		30.6	31.9	20.3	4	IL-17 C1
5	100.0	100.0	100.0	100.0		27.8	19.5	5	IL-17 C2
6	100.0	100.0	100.0	100.0	100.0		25.2	6	IL-17 D
7	100.0	100.0	100.0	100.0	100.0	100.0		7	IL-17 N
	1	2	3	4	5	6	7		

B

Percent Identity

Divergence	1	2	3	4	5	6	7	8	9		
1		45.2	15.9	8.8	10.0	21.5	20.9	9.9	9.2	1	IL-17 RA1
2	93.6		18.3	10.3	10.7	20.2	21.4	9.2	9.2	2	IL-17 RA2
3	100.0	100.0		13.1	15.3	18.6	18.6	8.9	11.3	3	IL-17 RB
4	100.0	100.0	100.0		34.7	10.8	9.1	8.8	11.3	4	IL-17 RC1
5	100.0	100.0	100.0	100.0		10.0	8.5	10.9	8.7	5	IL-17 RC2
6	100.0	100.0	100.0	100.0	100.0		29.7	8.4	8.7	6	IL-17 RD1
7	100.0	100.0	100.0	100.0	100.0	100.0		6.8	6.8	7	IL-17 RD2
8	100.0	100.0	100.0	100.0	100.0	100.0	100.0		26.2	8	IL-17 RE1
9	100.0	100.0	100.0	100.0	100.0	100.0	100.0	100.0		9	IL-17 RE2
	1	2	3	4	5	6	7	8	9		

Figure S1. Amino acid similarity of snakehead IL-17 (A) and IL-17R (B) proteins.
